# Contribution of frustules and mucilage trails to the mobility of diatom *Navicula* sp.

**DOI:** 10.1038/s41598-019-43663-z

**Published:** 2019-05-14

**Authors:** Lei Chen, Ding Weng, Chuan Du, Jiadao Wang, Shan Cao

**Affiliations:** State Key Laboratory of Tribology, Tsinghua University, Beijing, 100084 P.R. China

**Keywords:** Marine chemistry, Biosurfaces

## Abstract

The secreted mucilage trails of the diatom *Navicula* sp. in the process of motility were studied by scanning electron microscopy (SEM), transmission electron microscope (TEM), atomic force microscopy (AFM) and Raman spectra etc. Contrary to previous studies, force measurement was taken directly on the mucilage trails of live cells using the method of *in situ* force mapping by AFM. The retraction force curve presented an increased tip-substrate peak and a small saw-tooth pattern tip-mucilage peak. Especially, same measurements on various substrates with different surface energy revealed that the mucilage trails actually functioned as a medium increasing the adhesive force between the diatom and substrates, which is crucial to diatom’s adhesion and locomotion. In addition, the mechanical properties of mucilage trails were quite different from mucilage strands in the maximum adhesive force and the maximum polymer extension length. Raman spectra indicated the difference in compositions that both of the two kinds of mucilages had proteins and polysaccharide, but the mucilage strands contained some other components with C=O, —CH_2_— and —CH_3_ asymmetric and symmetric stretches. This research hammers out more precise information about mucilage trails which would be useful in terms of diatom motility and biofouling prevention.

## Introduction

Marine biofouling is a complex process that involves the adhesion, movement, growth and reproduction of marine organisms onto immersed artificial structures such as ship hulls, navigational instruments, aquaculture net cages and seawater intake pipes, causes problems such as increased drag, fuel consumption and instrument maintenance costs^1–5^. Marine immersion experiments have demonstrated that the first eukaryotic organisms to attach and establish initial biofilms are typically unicellular, photosynthetic and benthic diatoms^6–10^.

One of the unique characteristics of diatom cells is the highly ornamented silica cells shall (frustules) constructed like a Petri dish consisting of two halves jointed together with a girdle; and protoplasts are enclosed in these frustules where pore arrays are spanned^11^. Based on the characteristics and diversity of hierarchical frustules, diatom nanotechnology has intrigued collaborative researches in biology, physics, chemistry, material science, and engineering^12,13^. For instance, frustule structures inspired micro- and nanodevices designed^14^, such as artificial hinges and interlocking devices^15,16^, a click-stop mechanism^16^, micropumps^17^, and reinforced stabile contact^18^, dye-sensitized solar cells (DSSCs), nanostructured battery electrodes, and electroluminescent display devices^19,20^.

Highly motile diatom species, such as *Navicula* sp., can adhere strongly to hydrophobic surfaces and drift in response to fluctuations in the nutrient levels^21–23^. Diatom cells adhesion to different surfaces are assisted by the continual secretion of adhesive mucilage, a form of extracellular polymeric substance (EPS)^24–29^. For most raphid diatoms, two distinct adhesive mucilages are secreted^30^. One is a layer of slightly adhesive mucilage encases most of the cell surface, which called mucilage trails. The other one called mucilage strand is secreted from the passage of the raphe which is one or two slits through the valve face of mono-raphid or bi-raphid diatoms. Mucilage strands make the cell-substratum adhesion at the raphe providing the tractive driving force for diatom ‘gliding’, a special form of motility observed in raphid diatoms. Previous studies have suggested that as diatoms gliding over surfaces, the mucilage strands near the raphe are detached from the cell and left behind diatom ‘trails’, which eventually accumulate as initial components of biofilms^31,32^. Therefore, mucilage strands have a significant influence on biofilm formation processes.

Previous research have suggested that mucilage trails are relatively soluble, dispersive and not visibly detectable^24,32^, resulting in various difficulties in experimental observation. Tracer particles^32–34^, lectins and specific antibodies^35^ have been applied to mucilage trails as labels. However, these methods only enable detection, without providing topographical information. Chemical stains such as ‘Stains–all’ have been successfully used to dye trails, providing information on the topography of trails^36^, although some properties of the trails are altered by the staining process. Therefore, mechanical properties of mucilage strands should not be measured after chemical staining. By using AFM in ‘fluid tapping’ mode, Higgins *et al*., (2000) obtained topographical images of mucilage trails in a hydrated state, although the topography was not sufficiently detailed^31^. Due to the non-visibility of mucilage trails under optical microscopy, it is not possible to locate the correct position for cantilever tip placement to achieve the required adhesive force curve, resulted in difficulty in assessment of the mechanical properties of mucilage trails. Previous research^30,31,34^ has reported that it is preferable to utilize mucilage collected near the raphe to study mechanical properties, where the mucilage trails are thought to originate from, rather than trying to utilize the trails themselves. However, it remains unclear whether the mucilage trails are formed from the same mucilage that can be found near the raphe, whether physicochemical changes occur in mucilage after shearing and separation from the cell, or whether cells can change the composition of mucilage during ‘gliding’. Therefore, information on mucilage strands is still lacking and further investigation is essential to improve our understanding of diatom locomotion and biofouling.

The bending ability of frustule and locomotion is important to diatoms because they need to pass through a limited space without being buried by fresh sediment to arrive a better position with sufficient nutrient and light. In this research, chemical staining was used to obtain clear SEM images of dehydrated mucilage trails. Three-dimensional topography of the dehydrated mucilage trails was acquired by AFM. *In situ* force mapping on substrates in the vicinity of the passing diatom, making the direct measurement of mucilage trails’ adhesive forces possible. In addition, the compositions of mucilage trails were analyzed by Raman spectra, with the results of the mechanical properties and compositions of mucilage trails analyzed and compared to those of the mucilage strands.

## Materials and Methods

### Diatoms isolation and culturing

The unialgal diatoms *Navicula* sp. was purchased from institute of oceanology, Chinese Academy of Science, and the diatoms *Navicula* sp. were isolated from Jiaozhou Bay in Qingdao (36°N; 120°E), China. The culture medium was prepared based on the standard f/2 culture medium with additional Na_2_SiO_3_ (7.3 mg/L) to accelerate diatom activity. The culture medium was refreshed every 3 days and maintained under standard illumination conditions with a 12:12 h light/dark cycle (Philips fluorescent white bulb, 5,000 lux or 67.7 μE m^−2^ s^−1^), at 20 °C. For experimental diatoms were maintained under exponential growth conditions.

### Cleaning of diatom frustules

15 mL of hydrogen peroxide (H_2_O_2_, 35% aqueous solution) was added to cleaning the diatom frustules, and the mixture was heated to 90 °C for 4 h to ensure complete oxidation of the organic content. After 4 h sedimentation at room temperature, the upper layer of the mixture was removed and 5 mL of hydrochloric acid (HCl, 37% aqueous solution) was added, allowing reaction for 2 h. Then, the mixture with frustules was rinsed several times with DI-water, the upper layer was removed after 4 h of sedimentation each time. The bottom layer of the resulting solution was deposited onto a silicon chip and dried at room temperature. After complete drying, the cells were embedded and sectioned for TEM observation of the pores and nano-particles of frustules.

### Preparation of substrates

Five kinds of substrates including glass, silicon, polypropylene (PP), polyvinyl chloride (PVC) and polyglycine (Nylon) were selected because their different surface energies will create different adhesive conditions. All substrates were cut into 15 mm × 15 mm × 3 mm plates and polished. The surface energy and roughness of each substrate is listed in Table 1. Prior to experiments, all substrates were cleaned by sequential ultrasonic washing in acetone, ethanol and distilled water, for 20 min each.

**Table 1:** Surface energy and roughness of each substrate.

	Glass	Silicon	Nylon	PVC	PP
Surface energy (mJ/m^2^)	4400	1200	50.1	40.1	30.1
Surface roughness (nm)	24.8	10.5	63.2	55.4	96.2

### Chemical staining and preparation of diatoms for SEM

Chemical staining of the mucilage trails was performed by pipetting a few drops of suspended cells onto a silicon wafer placed in a plastic petri dish, then after allowing cells to settle for 10 minutes, about 20 mL of medium was added to the dish until the silicon wafer was fully immersed. The silicon wafer was systematically removed from the dish and 2 mL of Stains-All solution (0.1 g of “Stains All” [Sigma, St. Louis, MO, USA] in 100 mL of formamide) was added to the wafer surface for 5 minutes, for mucilage trail staining. The silicon wafer was then gently rinsed in sterile medium, a cover slip was placed upon the slide and sealed with wax. Cells were left to complete staining for 1 h, after which, the silicon wafer was dried by freeze-drying for 24 h. The samples were then viewed using a field emission gun scanning electron microscope (TESCAN LYRA3 FEG-SEM/FIB), with a focused ion beam operated at 15.0 kV.

### AFM topography scanning taken on dehydrated mucilage trails

During SEM observations, ring shape grooves were formed by the focused ion beam (FIB) near each observed area of mucilage trail, to ensure that the same mucilage trails were easily identifiable by optical microscopy using AFM (Asylum Research, Santa Barbara, CA, USA). The silicon wafer sample was glued to a glass slide and then mounted on the AFM magnetic sample stage, with observations performed in tapping mode, with a scan rate of 1 Hz and scan size of 5 μm.

### AFM force measurements

**Figure 1:**
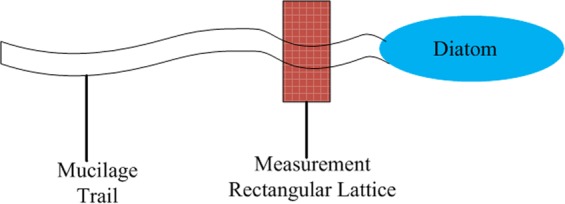
A schematic view of *in situ* forcing mapping by AFM.

**Figure 2:**
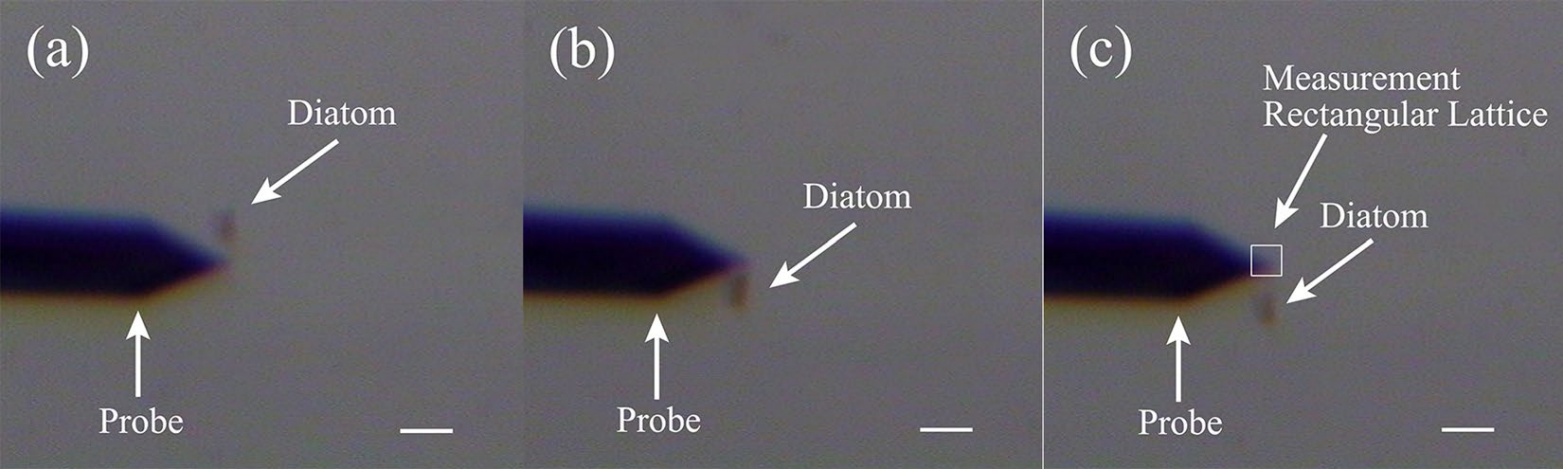
Optical microscope images of *in situ* forcing mapping by AFM. The diatom was moving from top to botom with the rectangular measurement lattice placed in the path directly behind the moving diatom. Scale bars: 20 μm.

AFM force measurements were firstly taken on clean substrates in both pure air and culture medium environments as comparative controls, prior to measurement on the mucilage trails in culture medium. The temperature and humidity were maintained at 25 °C and 10% RH, respectively. The probe used for force measurements, was an OMCL-AC240 silicon cantilever (Olympus Corporation, Japan) with a spring constant of 1.83 N/m which was determined using the thermal tune method^37^. Once mucilage trail force measurements had been taken, a fluid sample chamber was prepared by placing the substrate onto the bottom of a liquid-bath specially designed for use with an AFM. Live *Navicula* sp. cells were suspended in 1–2 mL of culture media, then the solution was transferred by pipette into the liquid-bath, then allowed to settle onto the substrate surface for 10–20 min. After settling, the liquid-bath was mounted under the AFM probe, which was used in conjunction with an optical microscope for easy visualization of diatoms. The probe was manually guided to be positioned in the path directly behind a moving diatom, with *in situ* force mapping assessed according to a 12 × 10 rectangular lattice (Figs 1 and 2) with 100 nm distance between measurement points. IGOR PRO molecular force probe software (Wave Metrics, Lake Oswega, OR, USA), was used to convert raw data into force versus separation distance curves. All force measurements are based on data from several different diatoms, using a new cantilever tip for each diatom.

### Raman spectra

Fluid sample chambers were prepared by placing an un-coated piece of 3 × 4 cm stainless steel substrate, onto the bottom of a glass petri dish. Live diatoms *Navicula* sp. cells were suspended in culture media and transferred to the petri dish by pipette, until the substrate was immersed. The chambers were then left undisturbed allowing cells to settle onto the substrate surface. After settling, the petri dish was mounted under the Raman microscope and Raman spectra were generated using a LabRAM HR 800 Raman microscope (Horiba Jobin Yvon) with a liquid-nitrogen cooled CCD) camera. An Ar ion laser with an excitation wavelength of 514 nm at 10 mw intensity was used, with an acquisition time of 30 seconds. All spectra were background-corrected and the spectral resolution was 1 cm^−1^. When measuring the mucilage trails following the diatom settling period (20 minutes), diatom motility by gliding on the surface of the substrate was visible by optical microscopy, with the Ar ion laser focused on the same rectangular measurement lattice as applied in AFM force mapping. When measuring mucilage strands, the diatom settling period was extended to 2 h, until large amounts of mucilage strands had visibly accumulated beside the motionless diatom, at which point the diatom was removed and the Ar ion laser was focused on mucilage strands.

## Results

### Observation of the hierarchical structure of diatom frustules

**Figure 3:**
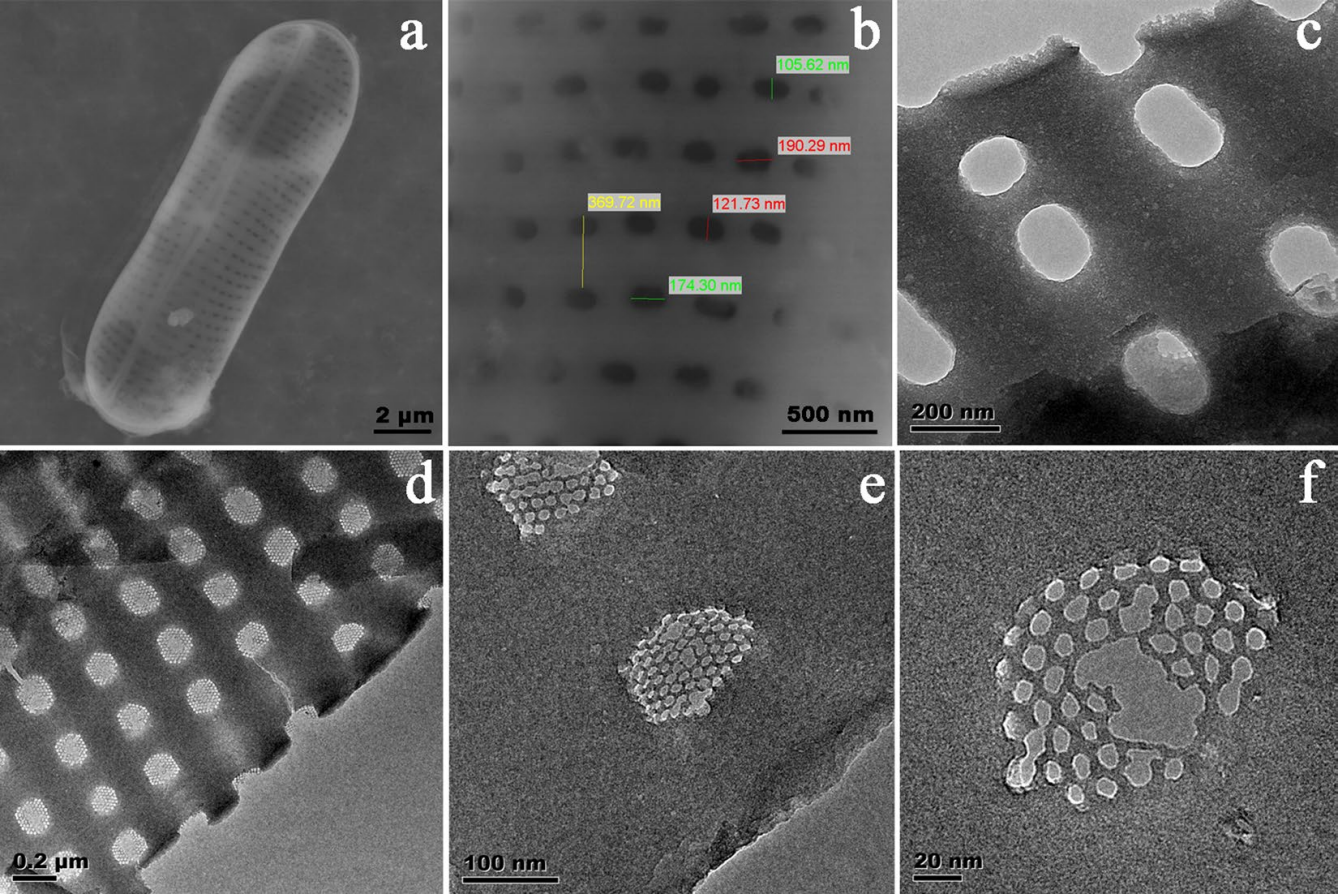
SEM and TEM images of frustules of diatom *Navicula* sp. (**a**) SEM image of overall image of a frustule. (**b**) SEM image of outer surface of the frustule (**c**) TEM image of outer surface of the frustule (**d**–**f**) TEM images of inner surface of frustule.

SEM and TEM images of the frustules of *Navicula* sp. is shown in (Fig. 3). The large-scale SEM images of the frustule (Fig. 3a,b) shows two-dimensional pore arrays. The pores are circular or elliptical with the size ranging from 100 ~ 200 nm. In the higher resolution TEM images (Fig. 3c–f), more detailed hexagonal holes with diameter of 10 nm can be observed in the other layer of the frustule. Therefore, it can be concluded that the *Navicula* sp. frustules have a hierarchical structure with two porous layers.

### Topography of dehydrated ‘trails’

**Figure 4:**
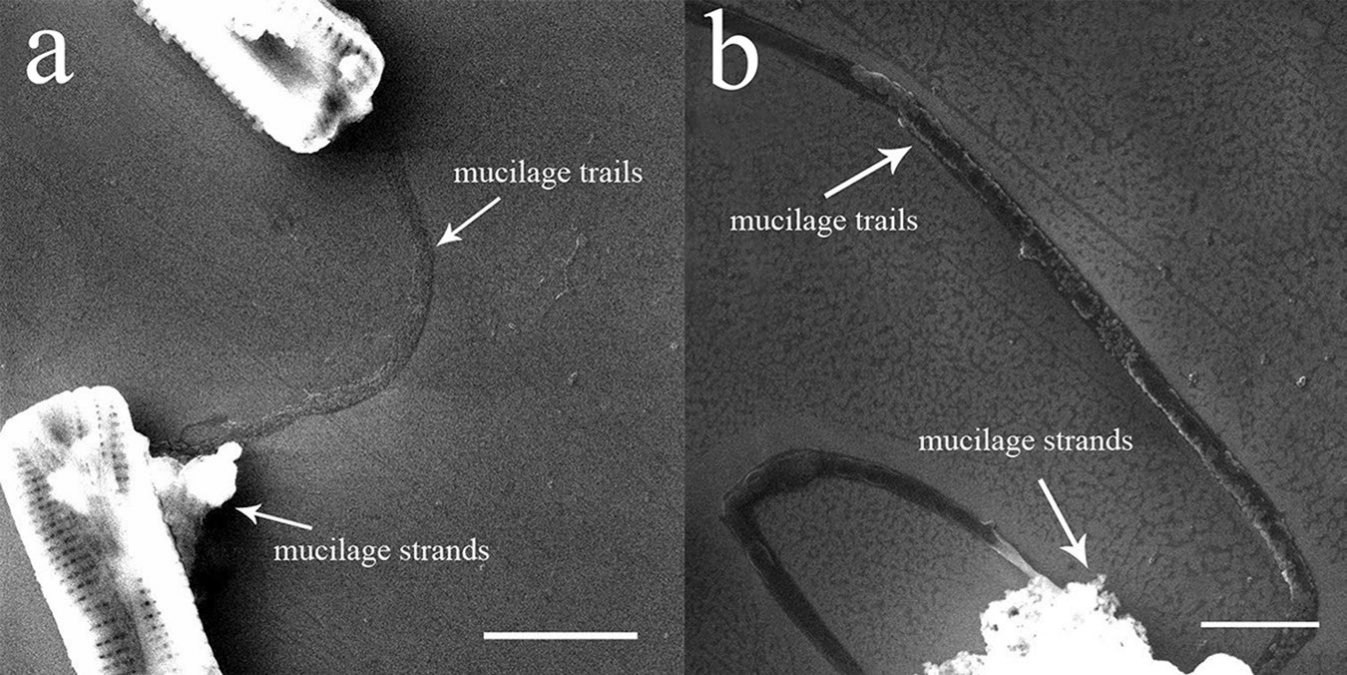
SEM images of dehydrated mucilage trails and mucilage strands. The mucilage trails were dark and left behind the diatom extending in straight or curved lines. The mucilage strands were bright, with disorderly accumulation observed beside the diatom. Scale bars: 5 μm (**a**), 2 μm (**b**).

SEM images of the gliding trajectories of diatoms are shown in (Fig. 4), with several visible mucilage trails left behind the diatoms on the silicon substrate, extending in straight or curved lines. The widths of the mucilage trails were approximately 400 nm. In addition, the gliding motion of diatoms was found to be flexible, with the ability to turn in successive 90° angles (Fig. 4b). Mucilage strands were also visible in the images, with a mass of strands found to be accumulated at the flank of the cell.

**Figure 5:**
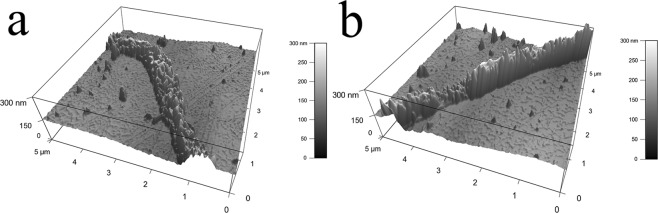
Three demensional topography of dehydrated mucilage trails by AFM. (**a**) was taken from the curved part of the mucilage trail in (Fig. 4a), while (**b**) was taken from the straight part of the mucilage trail in (Fig. 4b).

The three demensional topographical imges were taken by AFM in tapping mode, with the nano stucture of mucilage trails after dehydration shown in (Fig. 5). The average height of the trails was about 120 nm, with rough surfaces containing countless protrusions, which were likely to be formed during dehydration. The mucilage trail shown in (Fig. 5a) was wider and coarser than that in (Fig. 5b). It appears that mucilage was secreted at a constant flow rate, as when diatoms were gliding slowly, mucilage would accumulate to a greater degree, resulting in the trail being wider. In addition, when diatoms were gliding slowly the sheer speed was also slower, causing the outer surface to be coarser. Overall, non-uniform motion may result in the different topographies observed for mucilage trails.

### Force measurement by AFM

**Figure 6:**
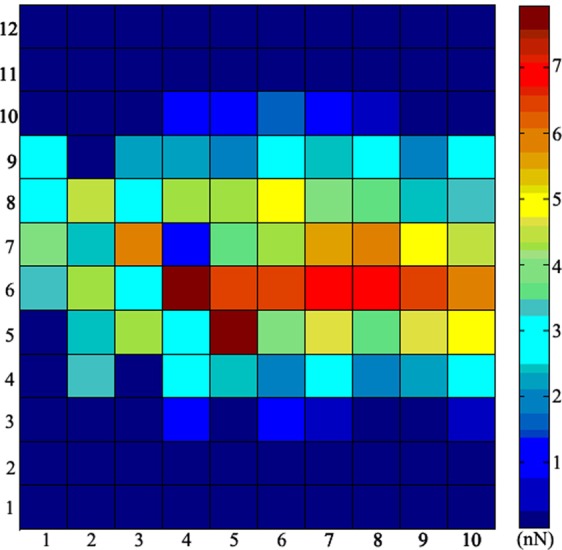
*In situ* force mapping by AFM on a glass substrate. Each square represents a maximum tip-substrate adhesive force, with the distance between centers of adjacent squares being 100 nm. The central force values are higher than those found at the top and bottom edges, with the highest observed value being 7.95 nN.

**Figure 7:**
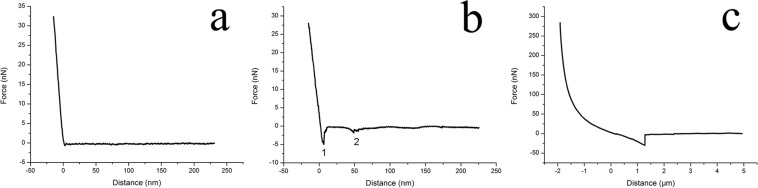
Characteristic adhesive (retraction) force curves on glass substrate in culture medium. (**a**) The force curve for clean substrate, with only a very small tip-substrate adhesive peak. (**b**) The force curve for mucilage trails, showing a notable tip-substrate adhesive peak and a saw-tooth patterned tip-mucilage adhesive peak. (**c**) The force curve for mucilage strands, showing only one very large tip-mucilage adhesive peak and non-linear variation at the contact area.

As mucilage trails were relatively soluble, dispersive and invisible by optical microscopy^24,32^, it was extremely difficult to measure force curves directly on mucilage trails. Instead, previous studies have measured the mucilage at the raphe area where the mucilage was thought to have been secreted from^24^, however, this requires that the diatom does not move during measurement. Unfortunately, with this observation method a significant assumption is made, that the composition of the mucilage does not change as it promotes the locomotion of diatom. Additionally, this method does not allow the adhesive forces between mucilage and substrates to be assessed. Moreover, many changes may occur in mucilage after shearing and separating from the cell. Therefore, in the present study *in situ* force mapping was adopted to obtain the real mechanical properties of fresh mucilage trails. The rectangle lattice was normal gliding direction of the diatom, ensuring it covered the full possible area where trails could be found. If there were mucilage trails, they should certainly be detected. Using this method, we got 120 force curves in every measurement. Analyzing these force curves and the maximum observed adhesive force between the AFM probe tip and the substrate, allowed a force map to be established, as shown in (Fig. 6). In comparison, the force between clean substrate and the probe tip in culture medium was also measured, with the results shown in (Fig. 6a). Meanwhile, as mucilage strands were easily visible by optical microscopy, they were directly measured by AFM, with the results shown in (Fig. 7).

The result in (Fig. 6) show a distinct area where the maximum adhesive forces were distinctly bigger than others. (Fig. 7a) shows that only a minor adhesive peak appeared when the probe tip separated from the clean substrate in culture medium, allowing this to be used as a baseline control. Therefore, any area with a maximum adhesive force larger than this control value, may be assumed to be part of the mucilage trail. The biggest value observed for the maximum tip-substrate adhesive force was 7.95 nN, with the values observed at the top and bottom edge being below 1 nN. As the distance between measurement points were 100 nm, the width of this trail was approximately 500–600 nm, which was wider than the dehydrated trails observed by SEM and AFM, which may be a result of the process of dehydration. The maximum adhesive forces found in the middle of the mucilage trails were larger than those detected on the edges, which may due to the mucilage in the middle was thicker than at the edges. It is noted that thicker mucilage trail areas were found to have a larger adhesive force. Detailed reason needs further detailed investigation.

**Figure 8:**
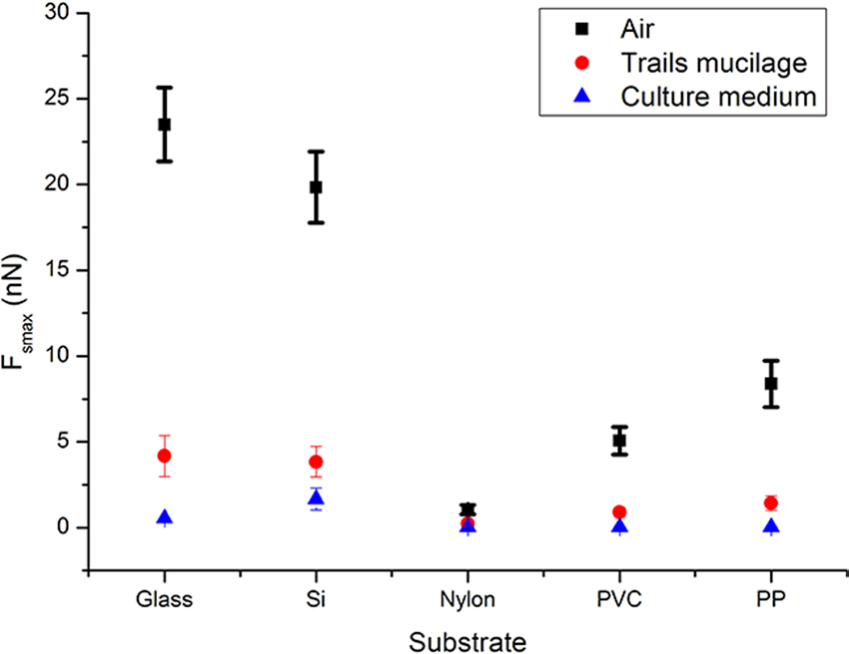
Comparison of the maximum adhesive force F_smax_ between cantilever tip and different substrates in different mediums. The black squares represent F_smax_ measured on clean substrates in the air. The red circles represent F_smax_ measured in mucilage trails on substrates in culture medium. The blue triangles represent F_smax_ measured on clean substrates in culture media. Error bars represent one standard deviation of calculation for 40 separate force curves (4 samples for each kind of material and 10 separate force curves for every sample).

According to the force map shown in (Fig. 6), a characteristic adhesive force curve with two adhesive force peaks could be detected and shown in (Fig. 7b). The first peak represents the maximum adhesive force between the AFM probe tip and the substrate around the mucilage trails (F_smax_), while the second peak represents the adhesive force between the AFM probe tip and the mucilage trails (F_max_). When comparing the adhesive force curve from (Fig. 7a) with that from (Fig. 7b), it was found that the adhesive force between probe tip and substrate is quite different from that between probe tip and mucilage trails. The reason could be when measuring the adhesive force of the mucilage trail, the probe tip was totally immersed in the mucilage trail. According to the XDLVO theory^38,39^ and Lifshitz theory of Van der Waals forces^40^, properties such as dielectric constant, refractive index, pH value, ionic strength and polymer states of the interaction medium, all significantly impact the adhesive force between the probe tip and substrate. In addition, adhesive force curves were measured on various substrates, including glass, silicon, polypropylene (PP), polyvinyl chloride (PVC) and polyglycine (Nylon) in air, culture medium and mucilage tails. The maximum adhesive forces observed between the probe tip and the substrate, are shown in (Fig. 8).

From (Fig. 8), it is clear that the maximum adhesive forces varied according to the different substrates and mediums. The F_smax_ measured on clean substrate decreased significantly from levels measured under air to under culture medium, however, the presence of mucilage trails on substrates under culture medium enhanced the F_smax_ to different extents depending on the substrate. Data shows that the larger the F_smax_ in the air, the larger the enhancement was with the presence of mucilage trails, suggesting that the material and surface properties also affect the adhesive forces of mucilage trails. In general, the mucilage trails were found to enhance the adhesive force between the diatom and all substrates tested in this experiment, although the extent of enhancement was related to the properties of the surface itself. As the surface energy of Nylon, PVC and PP were significantly lower than that of glass and silicon, it may be inferred that the extent of adhesive force enhancement by mucilage trails was smaller, on low surface energy materials.

**Figure 9:**
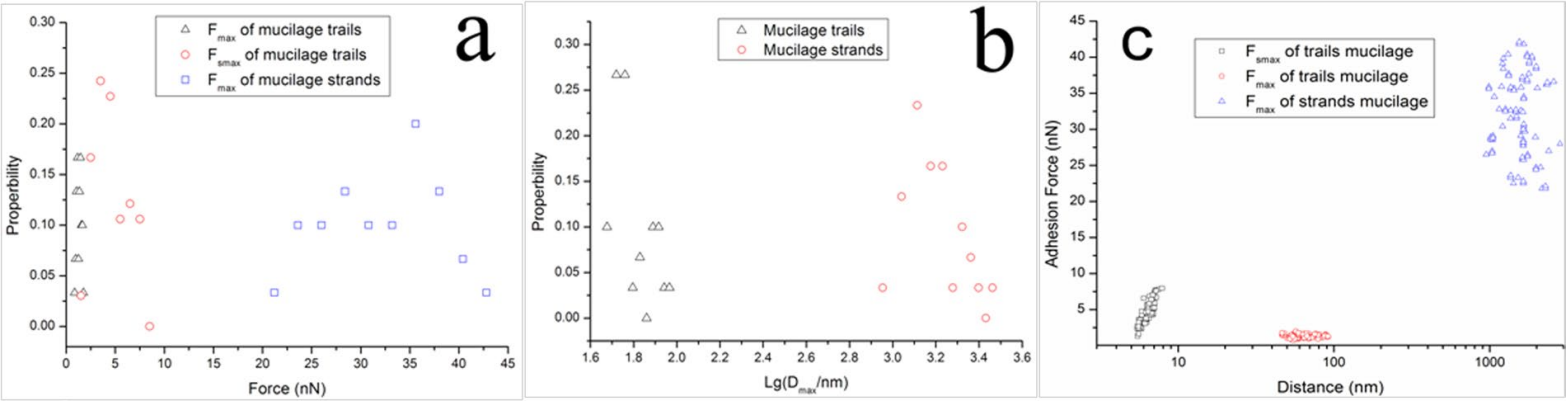
Probability distribution comparison figure of the maximum adhesive force F_smax_ and F_max_ (**a**) and the maximum polymer extension length D_max_ (**b**) of mucilage trails and mucilage strands. (**c**) The distribution of adhesion force and the distance of mucilage trails and mucilage strands.

In the retraction force curves established for mucilage strands (Fig. 7c), only one large adhesive peak was observed. In addition, the contact area of the retraction force curve for mucilage strands were non-linear, showing significant variation from the force curve established for mucilage trails in (Fig. 7b). The unique peak in the force curve for mucilage strands and the second peak in the force curve for mucilage trails, may be interpreted as the elastic response of a polymer chain after it was adsorbed to the probe tip and then stretched as the tip was withdrawn from the substrate. (Fig. 9a) depicts the probability distribution of F_smax_ and F_max_ of the adhesive forces of mucilage trails, as well as the F_max_ of the adhesive force curve for mucilage strands. The F_smax_ of mucilage trails were found to be between 2 nN–9 nN with a mean value of 4.51 ± 1.72 nN (mean ± SD; n = 90), while F_max_ values were between 0–2.5 nN with a mean value of 1.31 ± 0.24 nN (mean ± SD; n = 90). The F_max_ of mucilage strands were found to be between 20 nN–45 nN with a mean value of 32.1 ± 5.67 nN (mean ± SD; n = 90). (Fig. 9b) shows the probability distribution of the maximum polymer extension length D_max_, for both forms of mucilage. The D_max_ of mucilage trails was found to be between 45 nm–95 nm with a mean value of 62.8 ± 13.2 nm (mean ± SD; n = 90), while the D_max_ of mucilage strands was found to be between 0.8 μm–3.0 μm, with a mean value of 1.62 ± 0.48 μm (mean ± SD; n = 90). It was observed that both F_max_ and D_max_ of the mucilage strands, were much larger than those of the mucilage trails. Similarly, Higgins *et al*., (2002) performed AFM measurements on mucilage at the non-driving raphe and girdle area, at the surface of a stationary diatom *Craspedostauros australis*^30^. The F_max_ he got at the non-driving raphe was 2.09 ± 1.09 nN (mean ± SD; n = 105), while the F_max_ at the girdle area was 3.58 ± 1.97 nN (mean ± SD; n = 174), both of which being slightly larger than the F_max_ of mucilage trails established in the present study. The D_max_ of mucilage at the non-driving raphe and girdle area, were 357.8 ± 178.5 nm (mean ± SD; n = 90) and 725 ± 402.6 (mean ± SD; n = 164), respectively, which were significantly larger than the D_max_ established for the mucilage trails in the present study. As the diatom species were different, those comparisons are for reference only, however, a notably short D_max_ would be conducive to diatom motility by gliding.

**Figure 10:**
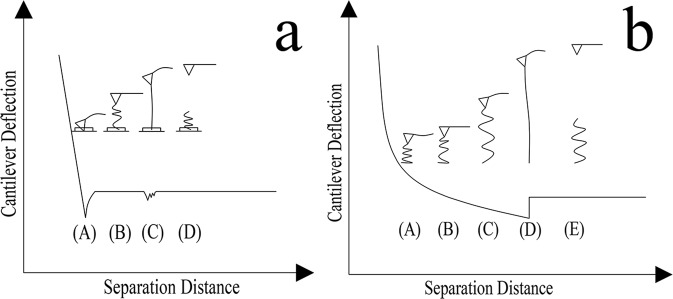
Schematic diagram of the relationship between the adhesive force distance curve and the extension of a polymer chain, in (**a**) mucilage trails and (**b**) mucilage strands.

To investigate how the cantilever tip interacts with the substrate and the mucilage polymer, a schematic diagram showing the relationship between the adhesive force distance curve and the polymer chain extension is presented in (Fig. 10). As shown in (Fig. 10a), before the cantilever tip detaches from the surface of the substrate in the mucilage trail, the force curve appeared to be linear. The cantilever tip then detached from surface at point A with a attached polymer chain. The polymer chain was stretched as the cantilever retracted from the surface to point B, generating a small undetectable cantilever deflection. When the polymer chain was further extended, the deflection of the cantilever became significant enough to be detected and the maximum adhesive peak appeared at point C. With further increase in tip-substrate distance, the applied force was large enough to make the polymer chain detach from the tip at point D. The interaction between cantilever tip and the polymer chain of mucilage strands was notably different from the interaction with mucilage trails. Initially, a tightly coiled and compressed polymer chain was attached to the cantilever tip at point A, resulting in a repulsive force after the cantilever tip detached from the substrate surface. With retraction of the cantilever, the polymer chain gradually stretched to a free and flaccid condition, with no force detectable at point B. When the polymer chain was stretched again with the cantilever’s retraction, a further increase was observed in the adhesive force at point C, which was distinctively different from point B in (Fig. 7a) and the findings reported by Higgins *et al*., (2003) where no obvious force was detected at point C^41^. The procedure from point D to E was followed the same pattern as from point C to D in (Fig. 10a).

**Figure 11:**
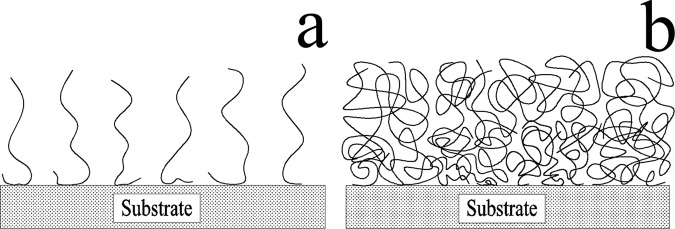
Schematic diagrams of the polymer states of mucilage trails and mucilage strands. (**a**) Polymers of mucilage trails were sparse and low-coiled with no entanglement. (**b**) Polymers of mucilage trails were dense and highly-coiled with high levels of entanglement.

The differences presented in the adhesive force curves of the two kinds of mucilage were probably caused by polymer state and composition. The different states of polymers^40^ are possibly the reason for the force observed at point C in (Fig. 10b). Since mucilage trails were thinner and smaller than mucilage strands, they contain a smaller amount of polymers, which implies that the polymers should be unperturbed in a low-coiled state, with reduced levels of entanglement between them (Fig. 11a). When these polymers were attached to the cantilever tip and stretched as the tip retracted from the surface, a small elastic downward force was detected, resulting in no adhesive force observed at point B in (Fig. 10a). As the polymer had a certain length, the low-coiled state also caused small maximum polymer extension length. However, mucilage strands accumulated more polymers to be gathered at a certain area. (Fig. 11b) shows the crowded, highly-coiled and interlaced polymer state of the mucilage strands. When a polymer in this state was attached to the cantilever tip and stretched as the tip retracted from the surface, it produced a large elastic force due to the interlacing of polymers. Consequently, the resultant force was large enough to be detected at point C in (Fig. 10b) and the highly-coiled polymer state also resulted in a bigger maximum polymer extension length.

### Raman spectra

Although the variation in elastic forces produced by different polymer states can partly explain the differences in the adhesive force curves between mucilage trails and mucilage strands, they do not account for the significant differences in F_max_ and D_max_. The F_max_ and D_max_ of mucilage strands were 24.5 times and 25.8 times bigger than those of mucilage trails, respectively. The types of the adhesive groups and the length of the polymers themselves were also found to be important factors.

**Figure 12:**
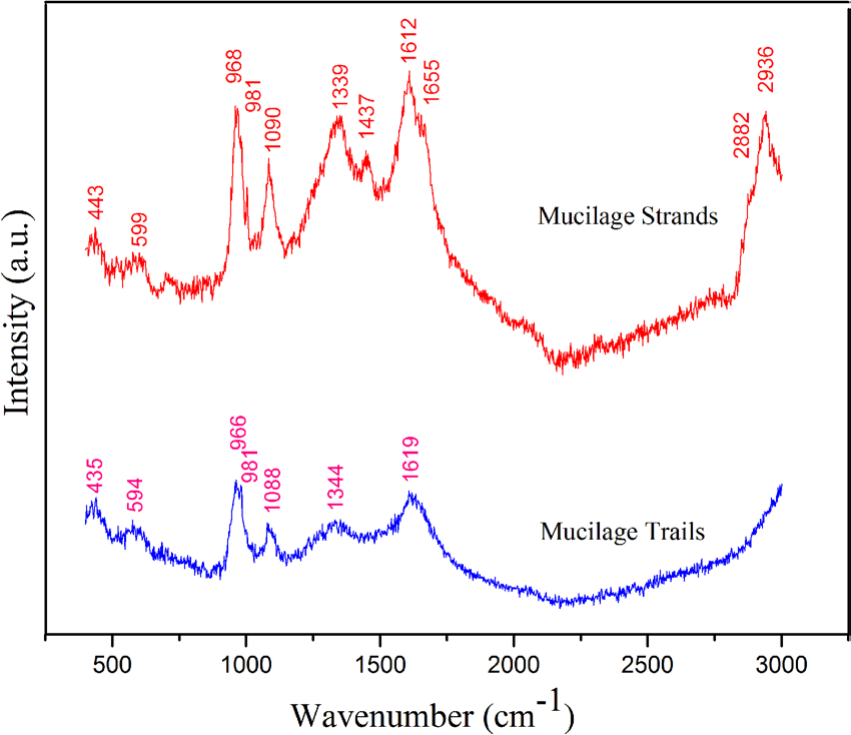
Raman spectra of the mucilage trails and mucilage strands, respectively.

Raman spectra were used to investigate the compositions of the mucilage trails and mucilage strands, and the spectra were shown in (Fig. 12). The results indicated that most of the compositions of the mucilage trails and strands were the same, such as 599 cm^−1^ and 594 cm^−1^ corresponding to phenylalanine, 1090 cm^−1^ and 1088 cm^−1^ agreeing with polysaccharide, 1612 cm^−1^ and 1619 cm^−1^ matching with tyrosine, both of the mucilage trails and strands had proteins and polysaccharide. However, the mucilage strands had some other bands like 1437 cm^−1^ corresponding to —CH_2_— deformation^42^, 1655 cm^−1^ corresponding to C=O, 2882 cm^−1^ and 2936 cm^−1^ matching with —CH_2_— and —CH_3_ asymmetric and symmetric stretches which could be carbohydrate. The bands detected on mucilage strands is much stronger than that of mucilage trails, because mucilage strands accumulate more polymers.

## Discussion

As *Navicula* sp. living at the water-sediment interfaces, it is important for them to find an ideal position with sufficient nutrients and lights to grow and reproduce. This research focus on the secreted mucilages, which reveal the locomotion ability of the diatom *Navicula* sp. Since proper chemical staining made the mucilage trails visible, allowing 2D and 3D topography to be obtained by SEM and AFM. The images clearly display the presence of mucilage trails extending in straight or curved lines, with an average height and width of approximately 120 nm and 400 nm, respectively. Compared with other observation methods such as tracer particles, lectins and specific antibodies, these results directly and clearly demonstrate the existence of mucilage trails. Although topography was measured in a dehydrated state and therefore some loss in continuity exists, these findings still provide much valuable information like dried surface appearance, detailed gliding trajectory and flexible locomotion behavior and so on.

Characteristic adhesive force curves of mucilage trails were obtained by AFM, with their mechanical properties analyzed and compared with mucilage strands. This is probably the first time that the mechanical properties of mucilage trails have been directly measured and the method of force mapping has been shown to be highly effective. These results confirm the existence of trails and give a precise distribution of mucilage trails adhesive forces, with the trails and trajectory also easily and clearly identified according to this distribution.

In the characteristic adhesive force curves, two main peaks are clearly distinct. The first peak represents the maximum adhesive force when the cantilever tip separates from the substrate, with a mean value of 4.51 ± 1.72 nN (n = 90). Generally, this force was small (below 1 nN) when measuring underwater, as the Van der Waals forces decrease significantly and the electrostatic force is mostly repulsive underwater. Therefore, when diatoms are moving underwater, mucilage trails may play a significant role as a medium to change the adhesive force between the diatom and substrate. Previous research has reported that diatom motility may rely on an actin-based cytoskeletal system, with trans-membrane proteins located just beneath the plasma membrane at the raphe^32^. These actin-based cytoskeletal systems drive trans-membrane proteins, which are linked to fibronectin or vitronectin in the mucilage trails to enable diatom motility via gliding^43^. According to this theory, no movement mechanisms require contact with the substrate and the trans-membrane proteins extend into the mucilage trails, pushing the diatom forward by propulsion. However, as mucilage trails increase the adhesive force to the substrate, it is assumed that the trans-membrane proteins may make contact with the substrates in the medium of mucilage trails, resulting in movement by a similar mechanism as a gecko ‘crawling’ on the substrate underwater. Geckos have been shown to move rapidly and defying gravity, using Van der Waals forces produced by the setal array on their toes^44^, although this form of motility would fail underwater, as Van der Waals forces are reduced. However, diatoms could ensure motility by secreting mucilage trails, replacing water as the medium and providing sufficient adhesive force between the trans-membrane proteins and substrate underwater. Direct evidence for this conjecture has not yet been found, although the large adhesive force observed between the substrate and mucilage trails, support it as a possibility.

Both the second peak of the mucilage trail characteristic adhesive force curve and the unique peak of the mucilage strand characteristic adhesive force curve, represent the elastic responses of their mucilage polymer chains. The shape of the force curves and the significant differences in F_max_ and D_max_, provide evidence of their variation. The F_max_ and D_max_ of mucilage strands were 24.5 times and 25.8 times bigger than in mucilage trails, respectively. Two factors responsible for this difference are the states and compositions of the polymers. In addition, when considering diatom movement behavior, as mucilage strands are used for attachment and adhesion when the diatom is stationary, a large adhesive force is required to fasten itself to the substrate. Conversely, mucilage trails are continually secreted and then sheared when the diatom is gliding, therefore, lower adhesive forces of the mucilage trails reduced resistance while moving. Similarly, the extension length of the mucilage trails was found to be much shorter than those of the mucilage strands, which is likely to allow easy detachment when moving.

The second peak of the characteristic adhesive force curve of the mucilage trails, was found to have a saw-tooth pattern with multiple peaks, reflecting the successive unbinding of polymer segments from the tip and the breakage of bonds formed within and between the polymers. Similar patterns also have been used to explain the high tensile strength and toughness of modular domains in natural adhesives^45,46^, titin protein^47^ and bone collagen^48^. However, the saw-tooth pattern observed in the present study is short and not distinctly obvious, as the mucilage trails are such thin layers that less polymers are interlaced and less bonds were formed between them. This phenomenon can also be observed in the unique adhesive peak of the characteristic adhesive force curve of the mucilage strands, but it is not obvious as the F_max_ is much bigger.

Diatom mucilages are complex and composed of multi-component materials. Many early staining and compositional studies have suggested that carbohydrates dominated diatom mucilage^24^, with later studies showing the co-occurrence of proteins and carbohydrates. Chiovitti *et al*.^29^ found carbohydrates, proteins and sulfate in mucilage from the diatom *Pinnularia viridis*, however, analysis often focuses on diatom surface mucilage and mucilage strands on the substrate. In the present study, Raman spectra detected phenylalanine, tyrosine and polysaccharides in both mucilage trails and strands, which is in accordance with the theory that diatom mucilage is composed of proteins and carbohydrates. The excrescent components C ═ O, —CH_2_— and —CH_3_, form asymmetric and symmetric stretches in mucilage strands and demonstrate the differences between the two kinds of mucilages. As mucilage trails are hard to collect due to their transparency and low level of secretion, improved methods are required for effective and accurate compositional analysis. Raman spectra does not provide exact compositions for the two kinds of mucilage, but results confirm they are significantly different, which is likely to be a contributing reason for the observed differences in F_max_ and D_max_.

Our previous work^49^ shows that the flexibility and the bending ability of the frustules of diatom *Navicula* sp. via *in-vivo* observations of cell locomotion, and micromanipulation indentation bending deformation were obtained to reveal that the frustules of the diatom are able to endure a great bending deformation without rupture. The finite element analyses further confirm that the diatom structure is more flexible in bending than the comparative structures of the same size or the same volume without pores. Adhesion was estimated from van der Waals force, showing that the generated underwater friction was able to maintain the deformation during cell locomotion. The observations of diatom locomotion and secreted mucilages indicate that some of the organisms can protrude out of the frustules from the raphe (~160 nm) to temporarily form one or two stalks^50^ or pseudopods^51^, contacting with substratum through the secreted mucilages and leaving pits (200~300 nm) in the gliding trails. The cell-substratum adhesion is required for diatom gliding. Micromechanical properties of diatom *Navicula pelliculosa* were semi-quantitatively measured by AFM, and regions with different mechanical properties were identified^52^. The elastic modulus varies from 7 to 20 GPa, from 20 to 100 GPa and from 30 to hundreds of GPa depending on the location of measurement. Also, the hardness ranged from 1 to 12 GPa, presenting a great spatial differences. Another loading test using calibrated glass microneedles was performed on three types of diatom cells^53^, showing that the frustules were remarkably strong and able to withstand mechanical stress corresponding to pressures of 100–700 tonnes/m^2^. An examination of fracture in the pennate diatom frustule showed that crack did not travel through, but around the minute (about 40 nm across) silica spheres forming the frustules^54^. This almost doubled the area of the fracture, thereby increasing the amount of energy necessary to break the frustule^53^. An AFM nanoindentation study indicated that mechanical properties were influenced by the pore size, pore distance, and porosity^55,56^. Simulation works^57,58^ reveal that nanoporous silica could be mechanically deformable and ductile, and the hierarchical structures were attributed to the exponential increase in toughness and defect-tolerance without introducing additional mechanisms or materials^58^. Hierarchical structures provide a path to transform a brittle material into a ductile system through alternating structural arrangement at the nanoscale^57^, results in high strength frustules to bear load and protect the protoplast as an armour^52,53^.

## Conclusion

By means of SEM, TEM, AFM, Raman spectra and other chemical biology techniques, the topography, mechanical properties and composition of the mucilage trails of diatom *Navicula* sp. have been described and analyzed in comparison with mucilage strands. These results provide a baseline for a better understanding of the characteristics of diatom mucilage and its function in diatom locomotive processes. In particular, the *in-situ* force mapping method by AFM enables measurement of mucilage trails force curves directly and determines their mechanical properties. In further studies, the mucilage trails detailed composition and specific function in diatom motility will be investigated. Moreover, a colloidal AFM probe will be used to investigate the adhesion between various particles and diatom mucilage. This research contributes significantly to the further study of diatom locomotion mechanisms and the prevention of biofouling.
